# Canagliflozin Inhibits Palmitic Acid-Induced Vascular Cell Aging In Vitro through ROS/ERK and Ferroptosis Pathways

**DOI:** 10.3390/antiox13070831

**Published:** 2024-07-11

**Authors:** Fang Wan, Xin He, Weidong Xie

**Affiliations:** 1 State Key Laboratory of Chemical Oncogenomics, Shenzhen International Graduate School, Tsinghua University, Shenzhen 518055, China; 18810205895@163.com (F.W.); hexingood56@163.com (X.H.); 2 Shenzhen Key Laboratory of Health Science and Technology, Institute of Biopharmaceutical and Health, Tsinghua University, Shenzhen 518055, China; 3 Open FIESTA Center, Shenzhen International Graduate School, Tsinghua University, Shenzhen 518055, China

**Keywords:** canagliflozin, senescence, ROS/ERK, ferroptosis

## Abstract

Vascular aging is one of the reasons for the high incidence of cardiovascular diseases nowadays, as vascular cells age due to various internal and external factors. Among them, high fat is an important inducer. Canagliflozin (CAN) is one of the SGLT2 inhibitors that has been shown to have cardiovascular protective effects in addition to lowering blood sugar, but the specific mechanism is not clear. This study first established a vascular aging model using palmitic acid (PA), then tested the effect of CAN on PA-induced vascular aging, and finally examined the mechanism of CAN’s anti-vascular aging via ROS/ERK and ferroptosis pathways. We found that CAN alleviates PA-induced vascular cell aging by inhibiting the activation of ROS/ERK and ferroptosis signaling pathways. This study reveals new mechanisms of lipid-induced vascular aging and CAN inhibition of vascular aging from the perspectives of ROS/ERK and ferroptosis pathways, which is expected to provide new ideas for the development of related drugs in the future.

## 1. Introduction

Aging has become a global public health challenge characterized by decreased cell function, increased cell death and damage leading to organ dysfunction, and the occurrence of many diseases, including cardiovascular disease and neurodegeneration [1]. The rate of the aging process depends on various factors and varies from person to person, and aging is the result of both exogenous and endogenous influences. In addition to age, the increase in free radicals, inflammation, and other pathological, physiological, and biochemical changes caused by internal and external factors can accelerate the process of cell aging [2,3].

Cardiovascular disease (CVD) is the leading cause of death worldwide. Among them, vascular aging, namely, degenerative changes in blood vessels, is one of the important risk factors for cardiovascular diseases. As is well known, age is one of the most important risk factors for vascular aging [4]. In addition, various internal and external environmental factors, such as diet, smoking, and hypertension, can also affect vascular aging in the body [5,6,7,8,9,10,11]. Vascular aging is the result of the aging of various vascular cells, such as endothelial cells and smooth muscle cells, which can lead to many pathological and physiological disorders [12], leading to cardiovascular diseases. Understanding the relationship between cardiovascular risk factors and vascular aging is critical for early prevention and the reduction in long-term cardiovascular risk. Inhibiting vascular aging caused by related factors is an important strategy to reduce cardiovascular disease [13].

Hyperlipidemia, also known as dyslipidemia, is mainly characterized by high triglycerides and hypercholesterolemia. Hyperlipidemia is a major risk factor for atherosclerosis. Free fatty acids (NEFAs) refer to non-esterified fatty acids in the serum. Research shows that high concentrations of NEFAs will lead to hyperfibrinogenemia, increased blood viscosity, decreased vascular fibrinolytic activity, and the deposition of fibrinogen in vascular walls, which will aggravate atherosclerosis [14,15,16]. Atherosclerosis is an important pathological factor behind cardiovascular diseases. Atherosclerosis is mainly manifested by lipid deposition, plaque formation, and vascular intimal inflammation [17]. Hyperlipidemia can lead to several disadvantages, including vascular stiffness and the aging of blood vessels [18]. High-fat-induced toxicity is one of the most important factors that contribute to vascular aging. Recently, breakthroughs have been made in the study of vascular aging in both cellular and molecular biology, particularly with regard to increased oxidative stress, chronic mild inflammatory responses, mitochondrial dysfunction, metabolic imbalance, impaired resistance to molecular stressors, genomic instability, cellular aging, epigenetic changes, the loss of protein homeostasis, and stem cell dysfunction [19].

Reactive oxygen species (ROS) play an important role in the aging process. As a crucial factor in vascular aging, ROS mediate signaling pathways that are significant mechanisms of vascular aging. ROS can serve as a key indicator for detecting the degree of vascular aging, and various signals of vascular aging are regulated by ROS [20].

Mitogen-activated protein kinase (MAPK) belongs to the Ser/Thr kinase family and is vital for complex cellular processes such as proliferation, differentiation, development, transformation, inflammatory response, and apoptosis by transmitting, amplifying, and integrating signals from various stimuli and triggering appropriate physiological responses. There are four subfamilies of MAPK, corresponding to four MAPK pathways: extracellular signal-regulated kinase (ERK), p38, c-Jun N-terminal kinases (JNK), and big mitogen-activated protein kinase 1/extracellular signal-regulated kinase 5 (BMK1/ERK5). ERK is regulated by many factors, among which the Raf-MEK-ERK pathway is the most representative MAPK signaling pathway, inducing cell growth and differentiation [21]. In addition, ERK is also involved in the regulation of cellular aging, and studies have shown that MAPK1-ERK1/2-MNK1/2-eIF4E (ERK1/2-eIF4E) is an important signaling pathway for regulating aging [22]. ERK can be activated by many factors, and it has been established that an increase in ROS can significantly promote the activity of ERK [23]. As age increases and various internal and external environmental stimuli occur, the ERK1/2 signaling pathway often exhibits higher activity, making it more susceptible to cellular aging.

Ferroptosis is a form of iron-dependent cell death, mainly mediated by the action of divalent iron or ester oxygenase, catalyzing the lipid peroxidation of highly expressed unsaturated fatty acids on the cell membrane, thereby inducing cell death [24,25]. Ferroptosis, as a form of death, is different from cell apoptosis, necrosis, and autophagy, involving different biological pathways and physiological processes [26,27]. Ferroptosis is dependent on intracellular ferrous ions, characterized by lipid peroxidation and iron deposition, ultimately leading to a decrease in the activity of glutathione peroxidase 4 (GPX4) and an increase in ROS, resulting in cell death [28,29].

Ferroptosis accelerates vascular aging, and eliminating the signal of vascular ferroptosis in age-related cardiovascular diseases is an important way to resist vascular aging [30,31,32]. During the process of ferroptosis, ROS can activate some transcription factors and protein kinases that promote ferroptosis while inhibiting some anti-ferroptosis signaling molecules, thereby regulating the process of ferroptosis [32,33]. Mitochondria participate in the regulation of these signaling pathways by producing signaling molecules such as ROS [31,34,35,36]. GPX4 is regulated by reduced glutathione (GSH). GPX4 acts as an important factor in the ferroptosis process, and its action is to interrupt lipid peroxidation [37,38]. An abnormal accumulation of intracellular iron can lead to lipid peroxidation and (GSH) depletion, which leads to GPX4 inactivation and ROS production through lipid peroxidation, resulting in cell ferroptosis. Recent studies have found that ERK activation also plays an important role in ferroptosis, and blocking the ERK pathway can inhibit ferroptosis and slow down the process of cell apoptosis [39,40].

Canagliflozin (CAN) is the first anti-diabetes sodium–glucose cotransporter 2 (SGLT2) inhibitor approved by the FDA in the USA. In addition to reducing blood sugar, it was found in clinical application that the drug can prevent cardiovascular diseases [41,42], but the specific molecular mechanism is not clear. Previous studies have shown that CAN has a certain anti-vascular-aging effect, but the specific molecular mechanism is not clear.

This study aims to create a cell model of palmitic acid (PA)-induced vascular aging through high-fat manufacturing and explore the effects of CAN on vascular aging and its specific molecular mechanisms from the perspectives of ROS/ERK and ferroptosis pathways. This provides potential research value and direction for the development of new drugs and mechanisms to resist and inhibit vascular aging.

## 2. Materials and Methods

### 2.1. Cell Culture and Treatment

Human umbilical vein endothelial cells (HUVECs) were purchased from the Cell Resource Center of the Chinese Academy of Sciences, Shanghai Institute of Life Sciences. The cells were placed in a carbon dioxide incubator (37 °C, 5% CO_2_) for cultivation. The cell culture medium consisted of 90% DMEM (Thermo Fisher Scientific/Gibco, Waltham, MA, USA) +9% fetal bovine serum (FBS, AUS-01S-02, Cell-box, Changsha, China) +1% dual antibiotics (penicillin/streptomycin) (Thermo Fisher Scientific/Gibco, USA). PA (Sigma-Aldrich, St. Louis, MO, USA) was gradually diluted with a sterilized 5% bovine serum albumin (BSA, Biofroxx, Heidelberg, Germany; g/mL, BSA/PBS) solution to obtain a PA stock solution (62.5 mM), which was stored at −20 °C for further determination. CAN, N-Acetylcysteine (NAC), and FR 180204 were purchased from MedChemExpress (MCE, Princeton, NJ, USA) and dissolved in DMSO. The cell administration groups are as follows: NOR is the normal control group, PA represents 0.3 mM PA-treated cells, and drug-treated groups include 0.1–0.5 μM CAN- and 1 μM FR180204/1 μM NAC/2.5 μM Fer-1-treated groups. Firstly, after the cells were seeded in culture wells for 12–24 h, the tested drugs indicated above were added to the respective groups according to the experimental design. Following that, except for the normal control group, both the PA control and drug-treated groups were induced with 0.3 mM PA. The normal or PA control groups were administered DMSO/BSA solutions with identical volumes. The final concentration of DMSO in each group was 0.1% (*v*/*v*), and the mass fraction of BSA was 5% (g/mL). This study used PA and drugs for modeling and drug treatment, and cells were incubated in a carbon dioxide incubator for 24 h. Corresponding samples were collected for further experiments.

### 2.2. Cell Viability—MTT Assay

Cell viability was assayed using MTT (3-(4,5-dimethylthiazol-2-yl)-2,5-diphenyltetrazolium bromide) (Coolaber, Beijing, China). MTT was prepared in sterile PBS as a 5 mg/mL stock solution, fully dissolved, and then filtered for sterilization using a 0.22 μm needle filter (PALL, New York, NY, USA). Cells were inoculated into 96-well plates. After 24 h of treatment, 20 μL of the MTT solution was added to each well, and the cells were further incubated at 37 °C for 4 h in the cell culture incubator. Then, the cell medium was removed, the formed formazan was dissolved with 200 μL of DMSO solution, and the absorbance at 490 nm was analyzed with an Epoch microplate spectrophotometer (Bio-Tek, Winooski, VT, USA).

### 2.3. Cell Cycle

The cells were inoculated in 6-well plates for 24 h. Then, the cell culture medium was carefully aspirated, and the cells were digested with trypsin to prepare a single-cell suspension. After centrifugation (1000× *g*, 5 min), the cell pellets were collected. The cell pellets were washed once with ice-cold PBS and collected by centrifugation. The cell pellets were gently mixed with 1 mL of pre-chilled 70% ethanol, stored overnight at 4 °C, and then centrifuged (1000× *g*, 5 min) to precipitate the cells, which were then washed once with ice-cold PBS. The staining solution consisted of 0.5 mL of staining solution, 10 μL of PI solution, and 10 μL of RNase A solution. Approximately 0.5 mL of the configured staining solution was added to each cell sample. The cells were gently mixed and resuspended, incubated for 30 min at 37 °C, shielded from light, and analyzed using a flow cytometer (Beckman Coulter Life Sciences, Brea, CA, USA).

### 2.4. Cellular ROS Assay—DCFH-DA Probe

Cells were inoculated in 96-well plates for 24 h. A DCFH-DA probe (Biyuntian, Shanghai, China) was diluted with a serum-free culture medium at 1:1000 to a final concentration of 10 μmol/L. The cell culture medium was removed, the diluted probe was added, and the cells were incubated at 37 °C for 20 min in a cell incubator. The medium was aspirated and washed three times with PBS, and the cells were observed and photographed under a fluorescent microscope (Leica Microsystems, Weztlar, Germany).

For ROS quantification experiments, the cells were inoculated in 12-well plates and subjected to the same treatment as above. After incubation was completed, the cells were digested in Eppendorf tubes using trypsin. The average fluorescence intensity of cells was measured using flow cytometry (Beckman, Brea, CA, USA).

### 2.5. Lipid ROS Detection

The C11 BODIPY 581/591 lipid peroxidation fluorescent probe (Mao-Kang, Shanghai, China) was used for the experiments. The cells were seeded at 1 × 10^4^ cells/well with 200 μL of cell suspension and cultured in 96-well plates for 24 h. After the completion of the culture, the DMEM medium was removed by aspiration and washed three times with PBS, and then 100 μL of the diluted fluorescent probe (1:1000 PBS) was added to each well and incubated for 1 h in a carbon dioxide cell incubator. The culture medium was removed by aspiration, and 200 μL of PBS was added to each well after washing three times with PBS. The cells were photographed by fluorescence microscopy or analyzed by flow cytometry.

### 2.6. Ferroptosis Detection

The FeRhoNox-1 ferroptosis fluorescent probe (Mao-Kang, Shanghai, China) was used for the experiments. First, 200 μL of the cell suspension was seeded in 96-well plates at 1 × 10^4^ cells/well. After incubation, the DMEM medium was removed by suction, cells were washed three times with PBS, and then 100 μL of the probe working solution (FeRhoNox-1 ferrous ion fluorescent probe stocking solution was diluted 1000-fold in PBS) was added to each well. After incubating for 1 h in a carbon dioxide cell incubator, photographs were taken by fluorescence microscopy and analyzed.

### 2.7. GSH Detection

This study used the GSH and GSSG detection kit (Biyuntian, Shanghai, China). Cells were seeded in 6-well plates at 1.5 × 10^5^ cells/well. After culture, the DMEM medium was removed by suction, and cells were washed once with PBS. Then, 500 μL of trypsin was added to each well for digestion for 30 s, cells were blown off by a pipetting gun, and cells were resuspended and washed twice with PBS after centrifugation. Then, according to the wet weight of the cells (10 mg of cells can be regarded as 10 μL), the reagent of the protein scavenger M in triploid volume was added according to the instructions of the kit, and the samples were quickly frozen and thawed twice using liquid nitrogen and a 37 °C water bath. The samples were then centrifuged at 12,000 rpm for 10 min at 4 °C to remove the supernatant for determination. The corresponding detection solution was added according to the instructions of each centrifuge tube kit, and then the absorbance at 412 nm was measured every 5 min by a microplate reader. Finally, the absorbance was compared with the standard curve to calculate the GSH content.

### 2.8. Western Blot

The cells were inoculated into 6-well plates and left for 24 h. Then, the culture dishes were placed on ice and washed twice with ice-cold PBS. Cell lysate (Biyuntian, Shanghai, China) reagent was added, and the cell lysate was collected into EP tubes. Then, the cell lysate was centrifuged at 12,000× *g* rpm for 10 min at 4 °C, and the supernatant was collected. Sample preparation was performed after the determination of the supernatant protein concentration with the Bradford assay (Beyotime Biotechnology, Shanghai, China). Subsequently, equal amounts of protein (30 μg/lane) were separated using 12.5% sodium dodecyl sulfate–polyacrylamide gel electrophoresis (Epizyme Biotech, Shanghai, China) and then transferred to nitrocellulose membranes (Pall, New York, NY, USA). The nitrocellulose membranes with the transferred proteins were then incubated and blocked for 2 h at room temperature using 5% skim milk (Epizyme Biomedical Technology Co., Ltd., Shanghai, China). Then, the blocked membranes were placed in the corresponding primary antibody solutions and incubated overnight at 4 °C. Primary antibodies (GPX4, xCT/SLC7A11, p53, p21, Phospho-p44/42 MAPK, P44/22 MAPK) were purchased from Cell Signal Technology (Danvers, MA, USA), while β-actin was sourced from Sigma-Aldrich. At the end of incubation with the primary antibody, the membranes were washed three times for 10 min each by shaking on a shaker using 1 × TBST. After washing, the membranes were placed in the secondary antibody solutions corresponding to the primary antibodies (Goat Anti-Mouse IgG or Goat Anti-Rabbit IgG, both purchased from Cell Signal Technology) and incubated for 2 h. At the end of incubation with secondary antibodies, the membranes were washed three times for 20 min each with 1 × TBST while shaking on a shaker and visualized using a luminescent solution (Thermo Fisher Scientific Inc., Waltham, MA, USA), and then the gray density of protein bands was analyzed using ImageJ (version 1) software.

### 2.9. Statistical Analysis

GraphPad Prism 8 software was used to analyze all experimental data, and the results are represented as mean ± standard deviation. One-way ANOVA was performed between groups.

## 3. Results

### 3.1. Canagliflozin Alleviates Cell Aging Induced by PA

**Figure 1:**
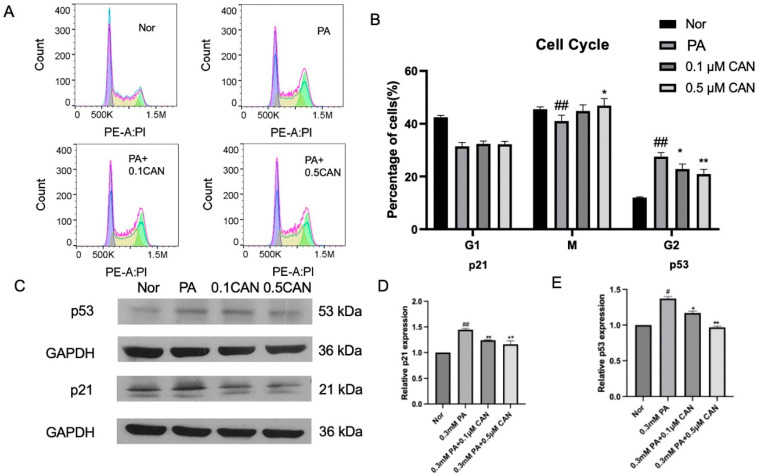
Canagliflozin alleviates cell aging of vascular endothelial cells induced by PA. (**A**) Cell cycle flow cytometry results; (**B**) cell cycle proportion; (**C**) p53 and p21 (aging-related indicators) protein WB results; (**D**) p21 protein change statistics; and (**E**) p53 protein statistical results (Nor represents normal control group, PA represents 0.3 mM PA-treated cells, 0.1CAN and 0.5CAN represent 0.1 μM and 0.5 μM CAN administration groups, ## represents *p* < 0.01 compared to Nor group, # represents *p* < 0.05 compared to Nor group, ** represents *p* < 0.01 compared to PA group, and * represents *p* < 0.05 compared to PA group, n = 5).

The cell cycle changes in HUVECs under different treatments were measured using flow cytometry. The results are shown in Figure 1A,B. The PA group showed a significant increase in G2-phase cells and cell cycle arrest compared to the normal group. After treatment with CAN, G2-phase cells were significantly reduced, and the decrease in the number of G2-phase cells was correlated with the concentration gradient of CAN, indicating that CAN can alleviate the cell cycle arrest caused by PA and delay cell aging.

The levels of p53 and p21 proteins, as cell cycle inhibitors, were measured, and the results are shown in Figure 1C–E. The PA group showed a significant increase in p53 and p21 proteins compared to the normal group. After treatment with CAN, the levels of p53 and p21 proteins were significantly reduced, and the decrease in p53 and p21 protein levels was correlated with the concentration gradient of CAN. This indicates that CAN can reduce the levels of the cell cycle inhibition proteins p53 and p21 induced by PA, thereby delaying cell aging.

This study demonstrated that CAN can reduce the increase in cell-aging-related indicators in vascular endothelial cells caused by fat accumulation, thereby delaying cell aging.

### 3.2. Canagliflozin Relieves Oxidative Stress and Lipid Peroxidation Caused by PA

**Figure 2:**
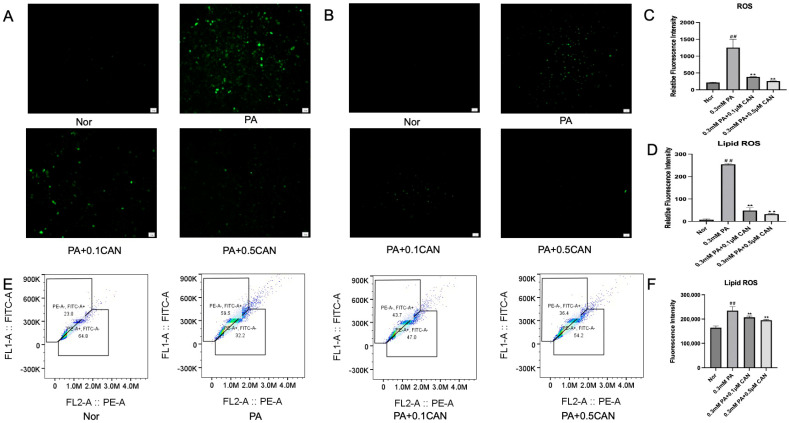
Canagliflozin relieves oxidative stress in vascular endothelial cells induced by PA. (**A**) Reactive oxygen species (ROS) fluorescence microscopy images; (**B**) Lipid ROS fluorescence microscopy image; (**C**) ROS image ImageJ statistical analysis results; (**D**) Lipid ROS image ImageJ statistical analysis results; (**E**) quantitative results of Lipid ROS flow cytometry; (**F**) statistical analysis of flow cytometry results (Nor represents normal control group, PA represents 0.3 mM PA-treated cells, 0.1 CAN and 0.5 CAN represent 0.1 μM and 0.5 μM CAN administration groups, ## represents *p* < 0.01 compared to Nor group and ** represents *p* < 0.01 compared to PA group, n = 5). The scale bar is 1 cm = 50 μm.

ROS are an important triggered factor affecting cell senescence. Firstly, we used the ROS fluorescence probe DCFH-DA to determine the changes in intracellular ROS levels in HUVECs under different treatments. The results are shown in Figure 2A,C. The PA group showed a significant increase in ROS levels compared to the normal group. After treatment with CAN, the intracellular ROS levels were significantly reduced, and the decrease in ROS levels was correlated with the concentration gradient of CAN. This indicates that CAN can alleviate the increase in ROS levels caused by PA, reduce intracellular ROS damage, maintain the cell state, and delay cell aging.

The Lipid ROS fluorescence probe was used to measure the changes in Lipid ROS in HUVECs under different treatments. The results are shown in Figure 2B,D. The PA group showed a significant increase in Lipid ROS levels compared to the normal group. After treatment with CAN, the intracellular Lipid ROS levels were significantly reduced, and the decrease in Lipid ROS levels was correlated with the concentration gradient of CAN, indicating that CAN can reduce the increase in Lipid ROS levels caused by PA and reduce the damage caused by intracellular Lipid ROS. In addition, we used the flow cytometry method to validate these results, and the results showed that CAN reduced the increase in Lipid ROS levels caused by PA (Figure 2E,F), which is consistent with fluorescence images.

Through the above experiments, it can be verified that CAN can reduce the increase in ROS levels and oxidative stress in vascular endothelial cells caused by fat accumulation, thereby delaying cell aging.

### 3.3. Canagliflozin Alleviates Cellular Aging by Reducing ERK Protein Phosphorylation Levels

**Figure 3:**
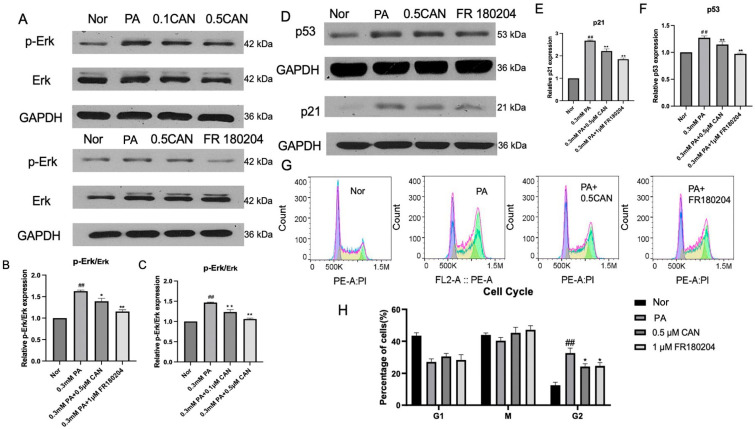
Canagliflozin alleviates cellular aging by inhibiting ERK protein phosphorylation. (**A**) Western blot detection of p-ERK and ERK proteins; (**B**) effect of FR 180204 on ERK protein phosphorylation; (**C**) effect of different concentrations of CAN on ERK protein phosphorylation; (**D**) detection of cell-cycle-related proteins using Western blot; (**E**) p21 protein level; (**F**) p53 protein level; (**G**) cell cycle flow cytometry results; (**H**) cell cycle ratio (Nor represents normal control group, PA represents 0.3 mM PA-treated cells, 0.1 CAN and 0.5 CAN represent 0.1 μM and 0.5 μM CAN administration groups, FR180204 represents the 1 μM FR180204 treatment group, ## represents *p* < 0.01 compared to Nor group, ** represents *p* < 0.01 compared to PA group, and * represents *p* < 0.05 compared to PA group, n = 5).

**Figure 4:**
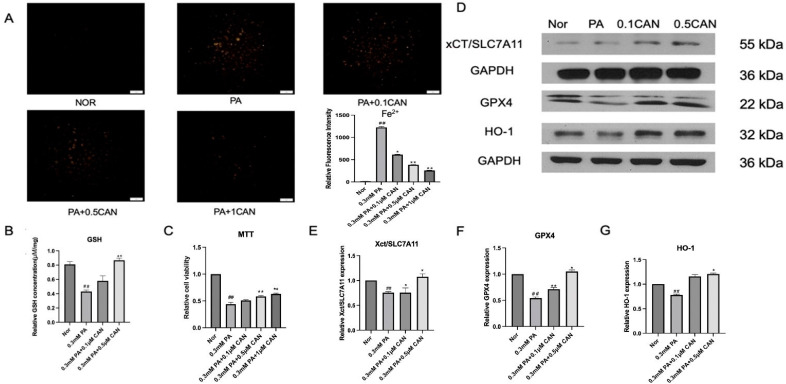
Canagliflozin inhibits ferroptosis in vascular endothelial cells induced by PA. (**A**) Western blot detecting p-Erk and Erk proteins; (**B**) reduced glutathione levels; (**C**) MTT cell viability detection; (**D**) detection of ferroptosis-related proteins by Western blot; (**E**) xCT/SLC7A11 protein level; (**F**) GPX4 protein level; (**G**) HO-1 protein level (Nor represents normal control group, PA represents 0.3 mM PA-treated cells, 0.1 CAN and 0.5 CAN represent 0.1 μM and 0.5 μM CAN administration groups, ## represents *p* < 0.01 compared to Nor group, ** represents *p* < 0.01 compared to PA group, and * represents *p* < 0.05 compared to PA group, n = 5). The scale bar is 1 cm = 50 μm.

Oxidative stress often causes an increase in cellular stress protein levels or activity. Here, we determined the ERK level. The Western blot method was used to analyze the ERK protein and its phosphorylation. The results are shown in Figure 3A,C and Figure 4B. The PA group showed a significant increase in p-ERK/ERK levels compared to the normal group. After treatment with CAN, p-ERK/ERK levels significantly decreased, and the decrease in p-ERK/ERK levels was correlated with the concentration gradient of CAN, indicating that CAN can alleviate the increase in p-ERK/ERK levels caused by PA. As shown in Figure 3B,C, after using the ERK phosphorylation inhibitor FR 180204, p-ERK/ERK significantly decreased, and FR 180204 can inhibit ERK protein phosphorylation.

Then, Western blotting was used to analyze the cell cycle inhibition proteins p53 and p21. The results are shown in Figure 3A,D–F. The PA group showed a significant increase in p53 and p21 proteins compared to the normal group. After treatment with CAN and FR 180204, the levels of p53 and p21 proteins were significantly reduced, indicating that CAN and FR 180204 can reduce the levels of the cell-cycle-related proteins p53 and p21 induced by PA by inhibiting ERK phosphorylation, thereby delaying cell aging.

The cell cycle changes in HUVECs under different treatments were measured using flow cytometry. The results are shown in Figure 3G,H. The PA group showed a significant increase in G2-phase cells and cell cycle arrest compared to the normal group. After treatment with CAN and FR 180204, G2-phase cells were significantly reduced, indicating that CAN and FR 180204 can alleviate the cell cycle arrest caused by PA by inhibiting ERK phosphorylation, thereby delaying cell aging.

Through the above experiments, it can be verified that ERK phosphorylation mediated cell senescence in vascular endothelial cells induced by fat accumulation and CAN may alleviate cell senescence by inhibiting ERK phosphorylation.

### 3.4. Canagliflozin Inhibits Ferroptosis Induced by PA

Recently, studies have emerged that indicate that ferroptosis plays a key role in contributing to cell senescence and viability. Firstly, we determined whether ferroptosis was seen in PA-induced vascular endothelial cells. We used the ferrous ion fluorescence probe FeRhoNox-1 to measure the levels of ferrous ions in HUVECs under different treatments. The results are shown in Figure 4A. The PA group showed a significant increase in ferrous ion levels compared to the normal group. After treatment with CAN, the intracellular levels of ferrous ions were significantly reduced, and the decrease in ferrous ion levels was correlated with the concentration gradient of CAN, indicating that CAN can reduce the increase in ferrous ion levels caused by PA and alleviate cell ferroptosis.

The GSH detection kit was used to determine the intracellular GSH levels in HUVECs under different treatments. The results are shown in Figure 4B. The PA group showed a significant decrease in GSH levels compared to the normal group. After treatment with CAN, the intracellular GSH levels significantly increased, and the increase in GSH levels was correlated with the concentration gradient of CAN, indicating that CAN can reduce the decrease in GSH levels caused by PA and alleviate cell ferroptosis.

The cell viability of HUVECs under different treatments was measured using MTT, and the results are shown in Figure 4C. The PA group showed a significant decrease in cell viability compared to the normal group. After treatment with CAN, the intracellular cell viability level significantly increased, and the increase in cell viability was correlated with the concentration gradient of CAN, indicating that CAN can alleviate PA-induced ferroptosis.

GPX4 is a key antioxidative protein, and its decrease is associated with increased lipid peroxidation and ferroptosis [43]. Heme oxygenase-1 protein (HO-1) is an antioxidative factor, and its decrease is associated with decreased antioxidative defense and increased ferroptosis [44]. Solute carrier family 7 member 11 (xCT/SLC7A11) is a cystine transporter and is associated with GSH synthesis for antioxidant defense. xCT/SLC7A11 has a well-established role in protecting cells from oxidative stress-induced ferroptosis [45]. Western blotting was used to analyze the ferroptosis-related proteins GPX4, HO-1, and xCT/SLC7A11, and the results are shown in Figure 4D–G. Compared with the normal group, ferroptosis-related proteins in the PA group were significantly decreased. After CAN treatment, ferroptosis-related protein levels were significantly increased, and the increases were correlated with the concentration gradient of CAN.

Through the above experiments, it can be verified that ferroptosis can be induced by fat accumulation in vascular endothelial cells; however, CAN can alleviate ferroptosis, thereby maintaining cell viability and protecting the cells.

### 3.5. Canagliflozin Alleviates Ferroptosis by Reducing ERK Protein Phosphorylation Levels

**Figure 5:**
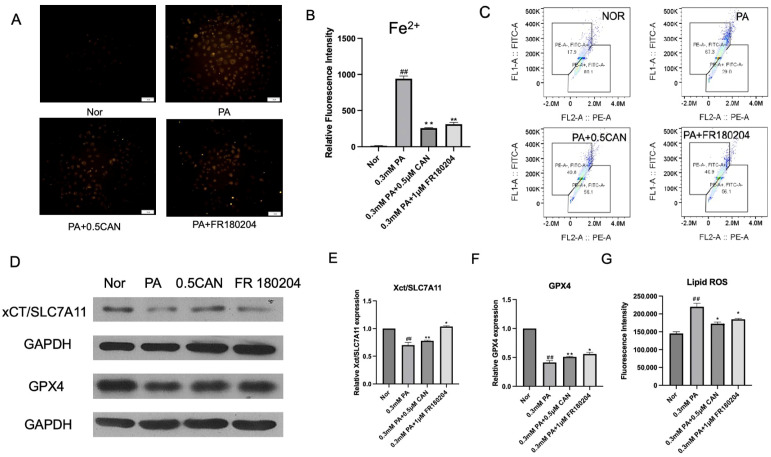
CAN alleviates ferroptosis by reducing ERK protein phosphorylation. (**A**) Ferrous ion staining in fluorescence microscopy images; (**B**) ImageJ analysis results of ferrous ion staining; (**C**) fluorescence microscopy images of lipid peroxidation (Lipid ROS); (**D**) detection of cell ferroptosis-related proteins using Western blot; (**E**) xCT/SLC7A11 protein level; (**F**) GPX4 protein level; (**G**) Lipid ROS experimental results analyzed through ImageJ statistical analysis (Nor represents normal control group, PA represents 0.3 mM PA-treated cells, 0.5 CAN represents 0.5 μM CAN administration group, FR180204 represents 1 μM FR180204 treatment group, ## represents *p* < 0.01 compared to Nor group, ** represents *p* < 0.01 compared to PA group, and * represents *p* < 0.05 compared to PA group, n = 5). The scale bar is 1 cm = 50 μm.

We have demonstrated that ROS levels, ERK activity, ferroptosis, and cell senescence increased in PA-induced vascular endothelial cells, and CAN showed some beneficial inhibitory effects. However, it remains unclear which factors are triggered or upstream, and it is critical to identify these factors. Firstly, we determined whether the ERK pathway affected ferroptosis. We used the ferrous ion fluorescence probe FeRhoNox-1 to measure the intracellular ferrous ion levels in HUVECs under different treatments. The results are shown in Figure 5A,B. The PA group showed a significant increase in ferrous ion levels compared to the normal group. After treatment with CAN and FR 180204 (a specific inhibitor of ERK), the intracellular ferrous ion levels were significantly reduced, indicating that CAN and FR 180204 might inhibit ERK phosphorylation, reduce the increase in ferrous ion levels caused by PA, and alleviate cell ferroptosis.

Using the Lipid ROS fluorescent probe C11 BODIPY 581/591, the changes in Lipid ROS in HUVECs under different treatments were measured. The results are shown in Figure 5C,G. The PA group showed a significant increase in Lipid ROS levels compared to the normal group. After treatment with CAN and FR 180204, the intracellular Lipid ROS levels were significantly reduced, indicating that CAN and FR 180204 can inhibit ERK phosphorylation, reduce the increase in Lipid ROS levels caused by PA, and reduce the damage caused by intracellular Lipid ROS.

Western blotting was used to analyze the ferroptosis-related proteins GPX4 and xCT/SLC7A11, and the results are shown in Figure 5D–F. Compared with the normal group, ferroptosis-related proteins in the PA group were significantly decreased. After treatment with CAN and FR 180204, the levels of ferroptosis-related proteins were significantly increased, indicating that CAN and FR 180204 could inhibit ERK phosphorylation and increase the levels of ferroptosis-related proteins induced by PA, which can reduce cell ferroptosis.

Through the above experiments, it can be verified that ERK phosphorylation mediated PA-induced ferroptosis in vascular endothelial cells, and CAN may inhibit the ferroptosis process by inhibiting ERK phosphorylation.

### 3.6. Canagliflozin Delays Cell Aging by Relieving Ferroptosis

**Figure 6:**
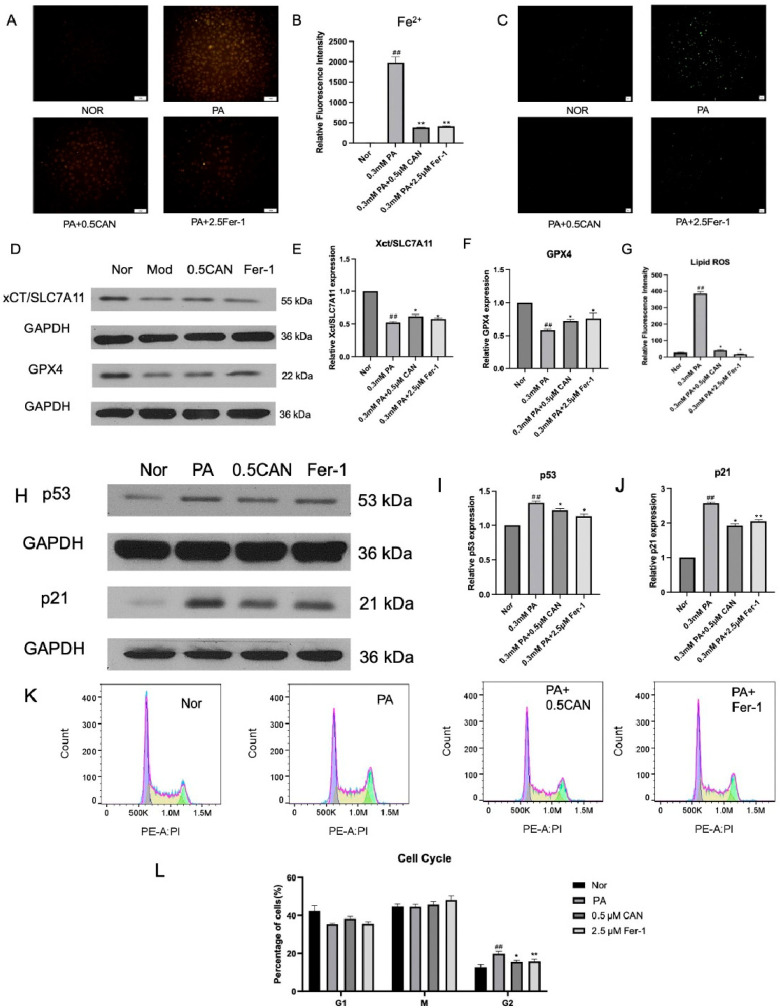
Canagliflozin antagonizes cell senescence by inhibiting ferroptosis. (**A**) ROS fluorescence microscope images; (**B**) ImageJ analysis results of ferrous ion staining; (**C**) fluorescence microscopy images of lipid peroxidation (Lipid ROS); (**D**) detection of cell ferroptosis-related proteins using Western blot; (**E**) xCT/SLC7A11 protein level; (**F**) GPX4 protein level; (**G**) Lipid ROS image ImageJ statistical analysis results; (**H**) Western blotting was used to detect p53 and p21 proteins; (**I**) p53 protein level; (**J**) p21 protein level; (**K**) cell cycle flow cytometry results; (**L**) cell cycle ratio (Nor represents normal control group, PA represents 0.3 mM PA-treated cells, 0.5 CAN represents 0.5 μM CAN administration group, Fer-1 represents 2.5 μM ferrostatin-1 treatment group, ## represents *p* < 0.01 compared to Nor group, ** represents *p* < 0.01 compared to PA group, and * represents *p* < 0.05 compared to PA group, n = 5). The scale bar is 1 cm = 50 μm.

As described above, we found that CAN inhibited ferroptosis and cell senescence. Although ferroptosis can affect cell senescence, whether the anti-aging effects of CAN in PA-induced vascular endothelial cells are associated with its inhibitory activity on ferroptosis remained unclear. In this study, we first used the ferrous ion fluorescence probe FeRhoNox-1 to measure the intracellular ferrous ion levels in HUVECs under different treatments. The results are shown in Figure 6A,B. The PA group showed a significant increase in ferrous ion levels compared to the normal group. After treatment with CAN and Fer-1, the intracellular ferrous ion levels were significantly reduced, indicating that CAN and Fer-1 can reduce the increase in ferrous ion levels caused by PA and alleviate cell ferroptosis.

Using the Lipid ROS fluorescent probe C11 BODIPY 581/591, the changes in Lipid ROS in HUVECs under different treatments were measured. The results are shown in Figure 6C,G. The PA group showed a significant increase in Lipid ROS levels compared to the normal group. After treatment with CAN and Fer-1, the intracellular Lipid ROS levels were significantly reduced, indicating that CAN and Fer-1 can reduce the increase in Lipid ROS levels caused by PA and reduce intracellular damage caused by Lipid ROS.

The Western blot method was used to analyze the ferroptosis-related proteins GPX4 and xCT/SLC7A11. The results are shown in Figure 6D–F. The PA group showed a significant decrease in ferroptosis-related proteins compared to the normal group. After treatment with CAN and Fer-1, the levels of ferroptosis-related proteins significantly increased, indicating that CAN and Fer-1 can increase the levels of PA-induced ferroptosis-related proteins, thereby reducing cell ferroptosis.

The Western blot method was used to analyze cell-cycle-related proteins p53 and p21. The results are shown in Figure 6H–J. The PA group showed a significant increase in p53 and p21 proteins compared to the normal group. After treatment with CAN and Fer-1, the levels of p53 and p21 proteins were significantly reduced, indicating that CAN and Fer-1 can reduce the levels of the cell-cycle-related proteins p53 and p21 induced by PA, thus delaying cell aging.

The cell cycle changes in HUVECs under different treatments were measured using flow cytometry. The results are shown in Figure 6K,L. The PA group showed a significant increase in G2-phase cells and cell cycle arrest compared to the normal group. After treatment with CAN and Fer-1, G2-phase cells were significantly reduced, indicating that CAN and Fer-1 can alleviate the cell cycle arrest caused by PA and delay cell aging.

Through the above experiments, it can be verified that ferroptosis induced by PA can promote cell senescence; however, CAN might inhibit cell senescence by inhibiting ferroptosis.

### 3.7. CAN Antagonizes ERK Activity, Ferroptosis, and Cellular Aging by Inhibiting ROS Production

**Figure 7:**
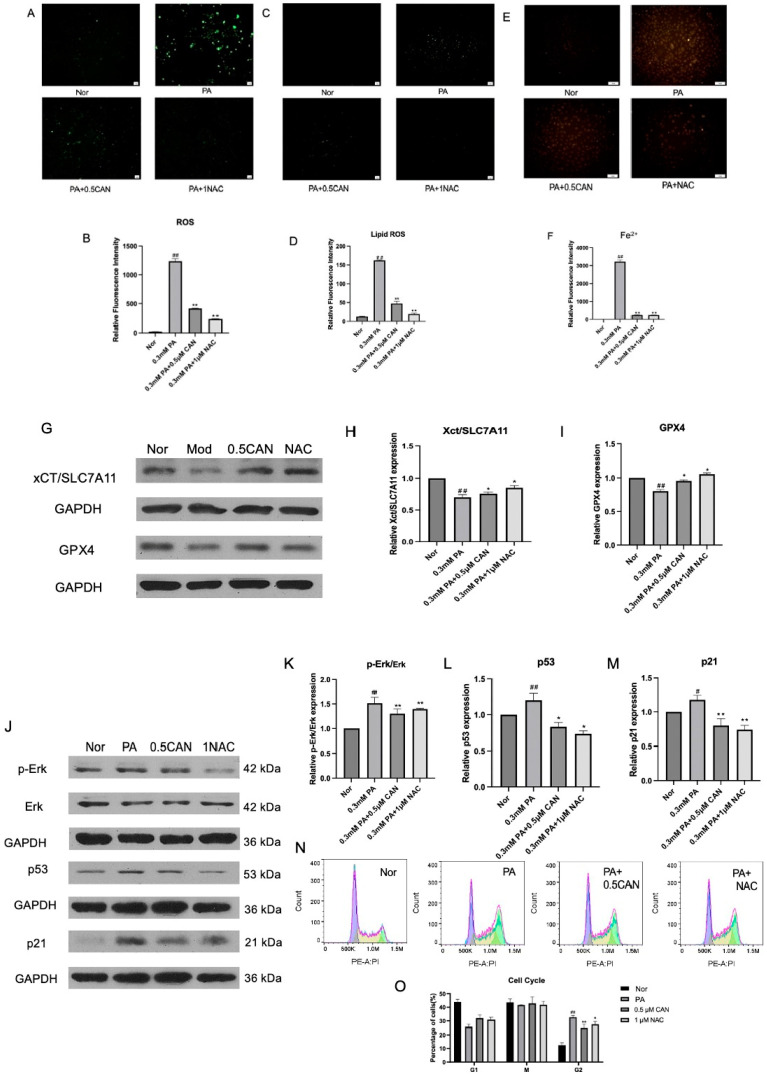
Canagliflozin antagonizes ERK protein phosphorylation, ferroptosis, and cell aging by reducing ROS levels. (**A**) ROS fluorescence microscopy image; (**B**) analysis results of ROS ImageJ; (**C**) Lipid ROS fluorescence microscopy images; (**D**) Lipid ROS ImageJ analysis results; (**E**) fluorescence microscopy images stained with ferrous ions; (**G**) ImageJ analysis results of ferrous ion staining; (**F**) detection of cell ferroptosis-related proteins using Western blot; (**H**) xCT/SLC7A11 protein level; (**I**) GPX4 protein level; (**J**) detection of p-ERK and ERK proteins, as well as cell-cycle-related proteins using Western blot; (**K**) p-ERK/ERK protein levels; (**L**) p53 protein level; (**M**) p21 protein level; (**N**) cell cycle flow cytometry results; (**O**) cell cycle ratio (Nor represents normal control group, PA represents 0.3 mM PA-treated cells, 0.5 CAN represents 0.5 μM CAN administration group, NAC represents 1 μM NAC treatment group, ## represents *p* < 0.01 compared to Nor group, # represents *p* < 0.05 compared to Nor group, ** represents *p* < 0.01 compared to PA group, and * represents *p* < 0.05 compared to PA group, n = 5). The scale bar is 1 cm = 50 μm.

Based on the experiments above, increased oxidative stress and ferroptosis are significantly increased in PA-induced vascular endothelial cells. As a result, we have confirmed that ROS can activate ERK, which, in turn, causes ferroptosis, and ferroptosis promotes cell senescence. Therefore, we believe that ROS may be a triggered factor, and the ROS/ERK pathway could contribute to ferroptosis and cell senescence. Additionally, we used NAC, a specific inhibitor of ROS, to eliminate intracellular ROS levels and observe how ROS affect ERK activity, ferroptosis, and cell senescence. Firstly, we used the ROS fluorescent probe DCFH-DA to measure the changes in intracellular ROS in HUVECs under different treatments, and the results are shown in Figure 7A,B. The PA group showed a significant increase in ROS levels compared to the normal group. After treatment with CAN and NAC, the intracellular ROS levels were significantly reduced, indicating that CAN and NAC can alleviate the increase in ROS levels caused by PA and reduce intracellular ROS damage.

Using the Lipid ROS fluorescent probe C11 BODIPY 581/591, the changes in Lipid ROS in HUVECs under different treatments were measured. The results are shown in Figure 7C,D. The PA group showed a significant increase in Lipid ROS levels compared to the normal group. After treatment with CAN and NAC, the intracellular Lipid ROS levels were significantly reduced, indicating that CAN and NAC can reduce the increase in Lipid ROS levels caused by PA and reduce the damage caused by intracellular Lipid ROS.

The ferrous ion fluorescence probe FeRhoNox-1 was used to measure the intracellular ferrous ion levels in HUVECs under different treatments. The results are shown in Figure 7E,F. The PA group showed a significant increase in ferrous ion levels compared to the normal group. After treatment with CAN and NAC, the intracellular ferrous ion levels were significantly reduced, indicating that CAN and NAC can inhibit ERK protein phosphorylation by reducing ROS levels, inhibit the increase in ferrous ion levels caused by PA, and alleviate cell ferroptosis.

The Western blot method was used to analyze the ferroptosis-related proteins GPX4 and xCT/SLC7A11. The results are shown in Figure 7G–I. The PA group showed a significant decrease in GPX4 and xCT/SLC7A11 proteins compared to the normal group. After treatment with CAN and NAC, the levels of GPX4 and xCT/SLC7A11 proteins significantly increased, indicating that CAN and NAC can inhibit ERK protein phosphorylation by reducing ROS inhibition and increase the levels of the PA-induced ferroptosis-related proteins GPX4 and xCT/SLC7A11, thereby reducing cell ferroptosis.

The Western blot method was used to analyze the ERK protein and its phosphorylation. The results are shown in Figure 7J,K. The PA group showed a significant increase in p-ERK/ERK levels compared to the normal group. After treatment with CAN and NAC, p-ERK/ERK levels significantly decreased, indicating that CAN can alleviate the increase in p-ERK/ERK levels caused by PA by reducing ROS levels.

Then, Western blotting was used to analyze the cell-cycle-related proteins p53 and p21. The results are shown in Figure 7J,L,M. The PA group showed a significant increase in p53 and p21 proteins compared to the normal group. After treatment with CAN and NAC, the levels of p53 and p21 proteins were significantly reduced, indicating that CAN and NAC can inhibit ERK phosphorylation by reducing ROS levels, reduce the levels of the cell-cycle-related proteins p53 and p21 caused by PA, and thus delay cell aging. The cell cycle changes in HUVECs under different treatments were measured using flow cytometry. The results are shown in Figure 7N,O. The PA group showed a significant increase in G2-phase cells and cell cycle arrest compared to the normal group. After treatment with CAN and NAC, G2-phase cells were significantly reduced, indicating that CAN and NAC can inhibit ERK phosphorylation by reducing ROS levels, alleviate PA-induced cell cycle arrest, and thus delay cell aging.

Through the above experiments, it can be verified that the increase in ROS level or oxidative stress induced by lipid accumulation can promote ERK phosphorylation and induce ferroptosis and cell senescence in vascular endothelial cells; however, CAN can reduce the cellular oxidative stress and lipid peroxidation levels, inhibit ERK activity, alleviate ferroptosis and cell senescence, and protect cells.

## 4. Discussion

Oxidative stress and inflammation mediated by ROS play an important role in the occurrence and development of aging [46,47]. The activation of ERK often mediates the occurrence of oxidative stress and inflammation [48]. The excessive accumulation of lipid substances in the blood vessel usually leads to toxicity, which is manifested as increased intracellular ROS, the oxidative stress response in cells, the activation of the MAPK pathway, and cell aging, damage, and even death [49,50]. p21 and p53 are cell-cycle-inhibitory proteins, which are often at high levels during cellular senescence [51,52]. In this study, we found that fat accumulation induced by PA significantly increased ROS levels and ERK activity, and we concluded that increased ROS and the activated ERK pathway may contribute to cell senescence induced by fat accumulation in vascular endothelial cells.

Ferroptosis is a kind of cell death specifically dependent on intracellular ferrous ions. The process of ferroptosis involves a change in transferrin SLC7A11, the accumulation of ferrous ions, a change in GPX4, a decrease in GSH, and an increase in Lipid ROS. In recent years, studies have found that ferroptosis plays a positive role in cell aging [30,31]. In this study, we found that PA significantly upregulated the levels of ferrous ions, Lipid ROS, and related proteins, suggesting that fat accumulation induced by PA promotes ferroptosis in vascular endothelial cells. We conclude that ferroptosis seems to play an important role in vascular aging induced by fat accumulation or lipotoxicity in vascular endothelial cells. Moreover, the correlation between ferroptosis and cell aging is a well-researched area [53]. Aging can be delayed by inhibiting ferroptosis [54]. Fer-1 is a classical ferroptosis inhibitor [55]. This study used Fer-1 as a ferroptosis inhibitor and found that Fer-1 not only reduced ferroptosis-related indicators but also had an effect on the cell cycle and alleviated cell senescence, which may be related to the inhibition of lipid peroxide accumulation and ROS levels caused by ferrous ion accumulation. The effect of CAN was similar to that of Fer-1 in inhibiting ferroptosis, and we hypothesized that the inhibitory effect of CAN on vascular cell aging may be related to the inhibition of ferroptosis.

Finally, considering that ROS, ERK, and/or ferroptosis can affect cell senescence, in this study, we used specific inhibitors to determine whether cell senescence induced by fat accumulation in vascular endothelial cells was associated with the mechanisms of ROS, ERK, and/or ferroptosis. In particular, we used NAC, a specific ROS scavenger, to perform experiments. The results showed that NAC could indeed reduce intracellular ROS and inhibit ERK protein phosphorylation. And, after the use of NAC, the phenomena of ferroptosis and cell aging were eased. The specific inhibition of ERK phosphorylation by FR180204 also inhibited ROS, indicating that ROS and ERK can interact with each other. The specific inhibition of ERK phosphorylation indeed attenuated ferroptosis and cell senescence, suggesting that ERK also mediated ferroptosis and cell senescence. In addition, we used a specific inhibitor of ferroptosis and found that ferroptosis is really involved in cell senescence induced by fat accumulation in vascular cells. So, it is very easy to see that the ROS/ERK pathway and ferroptosis are indeed involved in the vascular cell senescence induced by fat accumulation. ROS levels can be upregulated by increasing mitochondrial metabolism of PA or lipotoxicity [56]. Previous studies demonstrated that CAN can inhibit mitochondrial metabolism [57]. Through comparative studies, we found that CAN had similar effects on ROS production and suspected that CAN might reduce ROS levels by affecting PA mitochondrial metabolism or lipotoxicity, thereby inhibiting ERK phosphorylation, ferroptosis, and cellular senescence.

In this study, we found that CAN significantly inhibited the expression of senescence-related proteins such as p21 and p53 and affected the cell cycle in PA-induced vascular endothelial cells, suggesting that CAN may improve the appearance of cell senescence, which may be related to the inhibition of the ROS/ERK signaling pathway and ferroptosis-related proteins such as GPX4 and xCT/SLC7A11. However, the relationship between ROS and ERK pathways, ferroptosis, and aging caused by lipotoxicity, including other intracellular signaling pathways and related molecules, is very complicated, and they can connect with each other and crosstalk; determining whether the anti-aging effects of CAN on vascular cells specifically depend on the ROS/ERK pathway or ferroptosis process requires further research. On the other hand, this paper is limited to the cellular level, and animal experiments and clinical studies are needed to verify its findings in the future.

## 5. Conclusions

**Figure 8:**
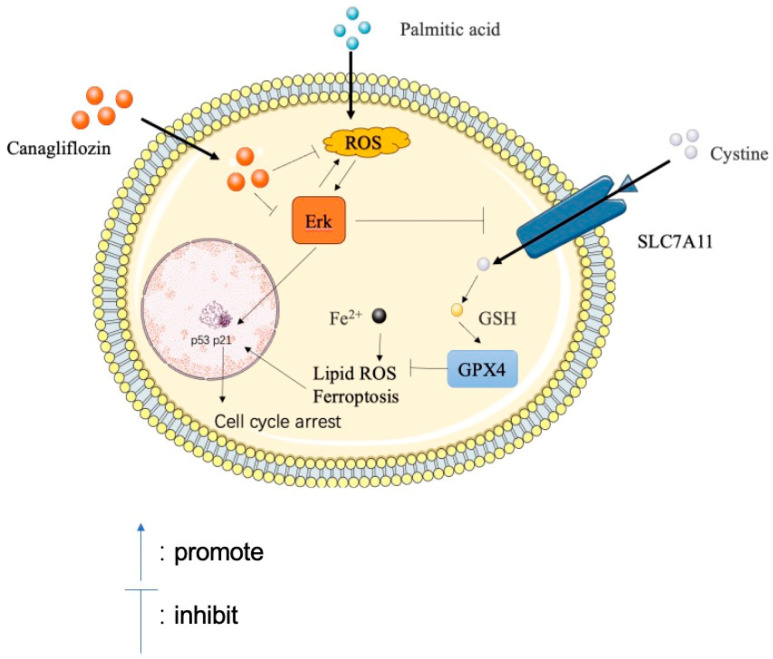
CAN inhibits PA-induced replicative cellular senescence via the ROS/ERK/ferroptosis pathway.

This study reveals that high-fat toxicity causes an increase in ROS levels and the activation of the ERK pathway in vascular endothelial cells. Simultaneously, high-fat toxicity upregulates Fe^2+^ and lipid oxidation levels, downregulates antioxidative factors (SLC7A11, GSH, and GPX4), and increases ferroptosis. Ultimately, high-fat toxicity leads to an increase in the levels of p53 and p21 proteins and causes cell cycle arrest and the aging of vascular endothelial cells. However, as a type of hypoglycemic drug, CAN exhibits new activity in inhibiting vascular aging, which may be related to the inhibition of ROS/ERK and ferroptosis pathways (Figure 8). This study also finds that through the classical inhibition of ROS/ERK and ferroptosis pathways, as well as comparative studies with CAN, targeting these signaling pathways can significantly inhibit high-fat-induced vascular cell aging. This study not only reveals the new effects of lipotoxicity on vascular aging from the perspectives of ROS/ERK and ferroptosis pathways at the cellular and molecular levels but also elucidates new mechanisms of vascular protection by CAN, providing new references for the development of anti-vascular-aging-related drugs in the future.

## Data Availability

The data presented in this study are available. If you need detailed data, you need to contact the corresponding author.
